# Editorial: RNA metabolism and DNA stability in the central nervous system (CNS): from aging to neurodegenerative disease

**DOI:** 10.3389/fmolb.2024.1385586

**Published:** 2024-03-07

**Authors:** Tangliang Li, Wenting Guo, Haibo Wang

**Affiliations:** ^1^ State Key Laboratory of Microbial Technology, Shandong University, Qingdao, China; ^2^ INSERM, UMR-S1118, Mécanismes Centraux et Périphériques de la Neurodégénérescence, NeuroStra Institute, CRBS, Université de Strasbourg, Strasbourg, France; ^3^ Department of Neurosurgery, Houston Methodist Research Institute, Houston, TX, United States; ^4^ Department of Neuroscience, Weill Cornell Medical College, New York, NY, United States

**Keywords:** RNA metabolism, DNA stability, PARP1, miRNA, Alzheimer’s disease

The faithful transmission of genetic information from the DNA to full functionalities of a cell safeguards healthy homeostasis in organisms. The integrity of DNA and RNA metabolism are two critical mechanisms in regulating cell vitality, disruptions of which are implicated in aging and neurodegeneration of the central nervous system (CNS) (De Conti et al., 2017; Welch and Tsai, 2022). In this Research Topic, a collection of findings on the Research Topic of posttranslational modifications of DNA repair proteins, miRNAs in spinal cord injury (SCI) and new techniques tackling Alzheimer’s disease (AD) pathologies have been published to reveal the importance of DNA stability and RNA metabolism in the brain.

Poly (ADP-ribose) polymerase 1(PARP1), as the major ADP-ribosyl transferase in mammalian cells, actively participates in various cellular processes, including DNA damage repair, chromatin remodeling, immunological response, etc. (Huang and Kraus, 2022). By associating with inflammation and autophagy dysfunctions, PARP1 activation is implicated in neurodegenerative diseases and the aging of brains (Mao and Zhang, 2022). Wang et al. employed the approach of HILIC affinity enrichment combined with MS analysis and identified 162 differentially methylated proteins in 293T cells upon ionizing radiation (IR) treatment (Wang et al.). In PARP1, at least 10 lysine residues have been identified to be mono-methylated and PARP1 K23 is the most prominently methylated residue following IR exposure. Their further investigation has revealed that PARP1 K23 methylation is not required for PARP1’s recruitment to DNA damage sites, but essential for the repair efficiency since cells expressing PARP1 K23A mutation exhibit a slower release of PAR and sustained γ-H2AX foci. Interestingly, PARP1 K23 mono-methylation is important for the repair of stalled replication forks. Although, Wang et al. did not investigate the roles of PARP1 K23 mono-methylation *in vivo*, it is conceivable that PARP1 K23 mono-methylation is important for mammalian brain development since other DNA repair factors, such as NBS1, involving in DNA replication regulation, play essential roles in human and mouse brain development.

miRNAs are a group of small non-coding RNA around 22 nt in length (Shang et al., 2023). They function through posttranscriptional regulation of gene expression and are pivotal in brain pathology, including neuronal damage and regeneration (Roy et al., 2022). Ghibaudi et al. conducted the sequencing of small RNAs after the SCI in mice, and found that the miR-7b-3q is significantly upregulated (Ghibaudi et al.). miR-7b-3q overexpression represses the expression of Wipf2, a positive neurite outgrowth regulator, thus promotes a plastic neural developmental phase at the expense of the axon growth. Meanwhile, miR-7b-3q overexpression inhibits apoptosis in cortical neurons. Ghibaudi et al.’s study not only revealed a panel of miRNAs actively responding to SCI, but also suggested a potential therapeutic strategy for SCI by targeting miRNA.

Alzheimer’s disease (AD), the most common cause of dementia, is a progressive neurodegenerative disorder with two pathological hallmarks, hyperphosphorylated Tau, and the formation and accumulation of amyloid plagues (Knopman et al., 2021). Tau’s phosphorylation is essential for its interaction with cellular filaments, including F-actin and microtubules, that regulates development and maturation of neurons (Johnson and Stoothoff, 2004). Alipour et al. conducted molecular dynamic (MD) simulation to analyze the effects of phosphorylation on three serine residues (Ser 262, Ser 285 and Ser 289) located on the short helical fragment of Tau, as well as to examine Tau’s inter-molecular interactions (Alipour et al.). The MD analysis indicated that the inter-molecular interaction of Tau, when all three sites are phosphorylated, is more stable and compact compared to the Tau with the sole phosphorylation on Ser 262. This study sheds lights on the relationships between the Tau phosphorylation and AD pathology. To tackle the question on directly quantifying the amyloid precursor protein (APP)-associated mitochondrial dysfunction in a single neuron, Niederschweiberer et al. combined the approach of NADH life imaging with the respiratory inhibitor treatment, which allows researchers to investigate the spatial and temporal dynamics of cellular biogenetics within a cultured neuron. Through this combined approach, they revealed that the overexpression of APP Swedish mutation (APPswe) leads to mild mitochondrial defects with reduced mitochondria respiration and ATP production. Furthermore, this research highlighted that within a neuron, mitochondria functions in soma are more susceptible to AD compared to those in the dendrites. This study established a single cell high-resolution strategy for *in vitro* characterization of mitochondrial dysfunctions contributing to neurological diseases.

Overall, scientific findings reported in current Research Topic not only reveal new mechanistic hints on DNA repair machinery and RNA regulation in neurobiology, but also provide new thoughts on utilizing novel tools, including bioinformatics and innovative technical approaches in exploring the cellular defects in degenerative brains.
